# Association between Soft Drink Consumption and Asthma among Qatari Adults

**DOI:** 10.3390/nu11030606

**Published:** 2019-03-13

**Authors:** Amna Al Ibrahim, Bushra Qamar, Sundus Fituri, Zoha Ali Akbar, Tamara Al-Abdi, Zumin Shi

**Affiliations:** Human Nutrition Department, Qatar University, P.O. Box 2713 Doha, Qatar; aa1305633@student.qu.edu.qa (A.A.I.); bq1408828@student.qu.edu.qa (B.Q.); sf1513326@student.qu.edu.qa (S.F.); za1404491@student.qu.edu.qa (Z.A.A.); tamara.alabdi@qu.edu.qa (T.A.-A.)

**Keywords:** soft drink, asthma, lung function, adults, Qatar Biobank

## Abstract

We aimed to examine the association between soft drink consumption and asthma and lung function among Qatari adults. In the cross-sectional study, we used data from 986 Qatari participants aged 20 years and above attending the Qatar Biobank Study. Usual consumption of soft drink was assessed using a food frequency questionnaire. Lung function was measured by spirometry and asthma was based on self-report. The associations between soft drink consumption and asthma and lung function were assessed using multivariable logistic and linear regression, respectively. In total, 65 participants out of 986 (6.6%) reported having asthma. A clear dose-response relationship between soft drink consumption and asthma was found. High soft drink consumers (≥7 times/week) were 2.60 (95% CI 1.20–5.63) times more likely to have asthma as compared to non-consumers. The association was partly mediated by BMI and inflammation. Diet soft drink consumption was positively associated with asthma (OR 1.12 (95% CI 1.02–1.23)) but not with lung function. Regular soft drink consumption was inversely associated with FEV1, but not with FVC. In conclusion, soft drink consumption is positively associated with asthma in Qatari adults. The association is partly mediated by obesity and inflammation. Limiting soft drink consumption should be taken into consideration for asthma prevention.

## 1. Introduction

Asthma is a common chronic pulmonary disease characterized by the clinical presentation of narrowing of airways due to chronic inflammation and bronchospasms [1]. It often overlaps with chronic obstructive pulmonary disease [1]. According to the Global Burden of Disease Study 2015, 0.4 million people died from asthma globally [2]. The prevalence of asthma was as high as 19.8% in school children in Qatar [3]. Among Qatari adults, the prevalence of asthma was around 9%. Asthma is a multifactorial disease with a complex interplay between genetics and the environment [4]. Several lifestyle and sociodemographic factors have shown to be associated with the risk of asthma, including smoking [5], obesity [6], sedentary lifestyle [4], and lower socioeconomic status [7]. 

There is evidence of an association between nutrition (including antioxidants, fat, and vitamin D) and asthma [8]. Over the past decade, several studies on dietary patterns and asthma have been published [9,10,11,12]. Most of these studies found associations between dietary patterns and asthma [10,12,13] or lung function [11,14]. 

Soft drinks are an important component of the Western lifestyle and diet. A positive association between soft drinks, especially sugar-sweetened beverages (SSBs), and asthma has been found in children and adults in different populations [15,16,17,18,19,20], mainly in Western countries. The link between soft drink and asthma has been hypothesized to be due to an increased level of inflammation [19,21,22,23], high intake of sodium benzoate, and high-fructose corn syrup [17,18,24]. However, as most of the existing studies used self-reported surveillance data without the actual measure of biomarkers, a limited number of studies have actually tested the hypotheses. 

The consumption of SSBs is high in the Middle East and North Africa (MENA) region [25,26]. In Qatar, 61.7% of students reported consuming at least one carbonated soft drink per day and more than 25% of the young adolescents consumed soft drinks three times or more per day [27]. 

There is no large-scale study assessing the relationship between soft drink consumption and asthma among adults in Qatar. Using data from Qatar Biobank Study, we aimed to (1) assess the association between soft drink consumption and asthma and lung function among adults; (2) assess whether inflammation mediates the association between soft drink consumption and asthma.

## 2. Materials and Methods

### 2.1. Study Design and Sample

Qatar Biobank recruited adults aged 18 years above who are Qatari nationals or those living in the country above 15 years. The detail of the study design was published elsewhere [28]. In brief, the study started in 2012 and aimed to recruit 60,000 participants. Up to December 2018, about 15,000 participants took part in the study. A self-administered questionnaire was used to collect sociodemographic information, lifestyle factors and dietary habits. Information on health condition, family history of disease, and medication use were collected during a nurse interview. Each participant was invited to have a health examination in the Qatar Biobank facility at Hamad Medical City. Body weight and height were measured by research nurses using a Seca stadiometer. Blood samples (60 mL) were taken and measured at the Qatar Biobank facility for a total of 66 biomarkers including C-reactive protein. The proportion of participants aged 18–19 was very small. In the current analysis, we included a random sample of 1000 Qatari participants aged 20 and above who answered the asthma-related question, completed the food frequency questionnaire and conducted a lung function test. Of the 1000 participants, 14 were excluded because they chose “prefer not to answer” to the asthma question. All participants gave written informed consent. The Qatar Biobank study was approved by the Institutional Review Board from the Hamad Medical Corporation Ethics Committee. The current analysis was approved under the IRB exempted category (Ex-2018-RES-ACC-0125-0069).

### 2.2. Outcome Variable: Asthma and Lung Function

Asthma was assessed by the questions during a nurse interview “Has a doctor told you that you have asthma?” and “How old were you when you first diagnosed with asthma?” The use of medication was also interviewed by the nurse. Thus, the claim of asthma was verified. 

Lung function was evaluated by Vitalograph Pneumotrac spirometry tests measuring forced vital capacity (FVC) and forced expiratory volume in one second (FEV1) [29]. The test was conducted at the Hamad Medical City.

### 2.3. Exposure Variable: Soft Drink Consumption

Habitual food intake was assessed by a self-administered food frequency questionnaire (FFQ). The FFQ included 102 food items. The FFQ asked participants to report their usual intake (never or rarely, 1–3 times per month, 1–3 times per week, 4–6 times per week, once per day, ≥2 times/day) of four types of drinks including regular soft drink, energy drink, diet soft drink, and fruit juice. 

In the analysis, the frequency consumption of soft drink was recoded to times/week based on the following rules: never or rarely (0), 1–3 times per month (0.5), 1–3 times per week (2), 4–6 times per week (5), once per day (7), and two or more times per day (14). We calculated total soft drink consumption by summing up the frequency intake of regular soft drinks, diet soft drinks, and energy soft drinks. Fruit juice was not included in the calculation of total soft drink and treated separately. Habitual fruit and vegetable intake was also recoded into times/week in the analysis.

### 2.4. Covariates

The following variables were treated as covariates: age, gender, education (low: primary and secondary school; medium: technical or professional school; high: university and postgraduate degree), leisure time physical activity level (metabolic equivalent of task (MET), recoded as tertiles), smoking (non-smokers, ex-smokers and current smokers). Overweight was defined as a BMI of 25.0 to 29.9 kg/m^2^; obesity was defined as a BMI of ≥30 kg/m^2^. C-reactive protein was used as an indicator of inflammation [30] and recoded into <6 mg/L or ≥6 mg/L. In the analyses, we did not exclude participants with missing values of BMI and CRP. When BMI and CRP were treated as categorical variables in multivariable models, missing values of BMI and CRP were assigned to a separate category. 

### 2.5. Data Analyses

Total soft drink consumption was recoded into four groups: non-consumers, ~1 time/week, 1–6 times/week, and ≥7 times/week. The chi-squared test was used to compare differences between groups for categorical variables and ANOVA for continuous variables. Logistic regression was used to assess the association between soft drink consumption and asthma. A set of four models were used: model 1 adjusted for age and gender; model 2 further adjusted for smoking, education, physical activity and intake of fruits and vegetables; and model 3 further adjusted for BMI (normal, overweight and obese) and CRP. Model 3 was conducted to assess whether the association between soft drink consumption and asthma was mediated by obesity and inflammation. The fourth model excluded those who were first diagnosed with asthma below the age of five years as the etiology of childhood asthma may be different from adult asthma. The association between soft drink consumption and lung function was assessed using a linear regression following the same adjustment strategy as above. The associations between soft drink, fruit juice and intake of fruit and vegetable were assessed using linear regression and presented graphically. In sensitivity analyses, multiple imputation was conducted to handle missing values of covariates using *mi* command in STATA 15 (Stata Corporation, College Station, TX, USA). As the main findings remained the same, we only presented the results from analyses without using multiple imputation. Multiplicative interaction between gender and soft drink consumption was tested by adding the product term of gender and soft drink consumption in the multivariable model. As there was no significant interaction between gender and soft drink, we decided not to present gender specific results (data not shown). All the analyses were performed using STATA 15 (Stata Corporation, College Station, TX, USA). Statistical significance was considered when *p* < 0.05 (two sided). 

## 3. Results

### 3.1. Sample Description

Among the 986 participants, 498 (50.5%) were male and 488 (49.5%) were female. While 36.1% of the participants did not consume soft drinks, 13.2% had soft drinks ≥7 times per week. The mean frequency intake of fruit juice, regular soft drink, diet soft drink and energy drink were 2.7, 1.7, 0.6, and 0.2 times per week, respectively. In total, 65 (6.6%) participants reported having asthma. Table 1 shows the sample characteristics across levels of soft drink consumption. Soft drink consumption was inversely associated with age but positively associated with smoking. The average age for non-consumers was 45 years as compared with 33 years among daily consumers. The prevalence of asthma was 5.9% and 11.5% among non-consumers and high consumers (≥7 times/week) of soft drink, respectively. Overall, leisure time physical activity was low. A decreasing trend of physical activity across soft drink consumption levels was observed, although not statistically significant.

Fruit juice consumption was positively associated with the consumption of fruits and vegetables (Figure S1). An increase of fruit juice consumption of 1 time/week was associated with an increase of fruit and vegetable consumption of 0.71 (95% CI 0.58–0.84) time/week and 1.05 (95% CI 0.73–1.38) time/week, respectively. Diet soft drink consumption was positively associated with fruit consumption but not vegetable consumption.

### 3.2. Association with Asthma

Soft drink consumption was positively associated with asthma (Table 2). After adjusting for age and gender, compared with non-consumers, high consumers (≥7 times/week) had an odds ratio (OR) for asthma of 2.51 (95% CI: 1.17–5.36). In the fully adjusted multivariable logistic regression (model 2), the ORs (95% CI) for asthma were 1.23 (0.61–2.51), 1.00 (0.48–2.08), and 2.60 (1.20–5.63) across non-consumers, ≤1 time/week, 1–6 time/week, ≥7 times/week of soft drink consumption, respectively. Compared with the models with and without adjustment of BMI and CRP, the OR for asthma among the daily consumers of soft drink changed 10.0% (from 2.60 to 2.33). After further excluding those with a diagnosis below the age of five, there was a clear dose response relationship between soft drink consumption and asthma (*p* for trend 0.010). Those who consumed soft drink ≥7 times/week were 2.64 (95% CI: 1.10–6.36) times more likely to have asthma as compared to non-consumers. 

**Figure 1:**
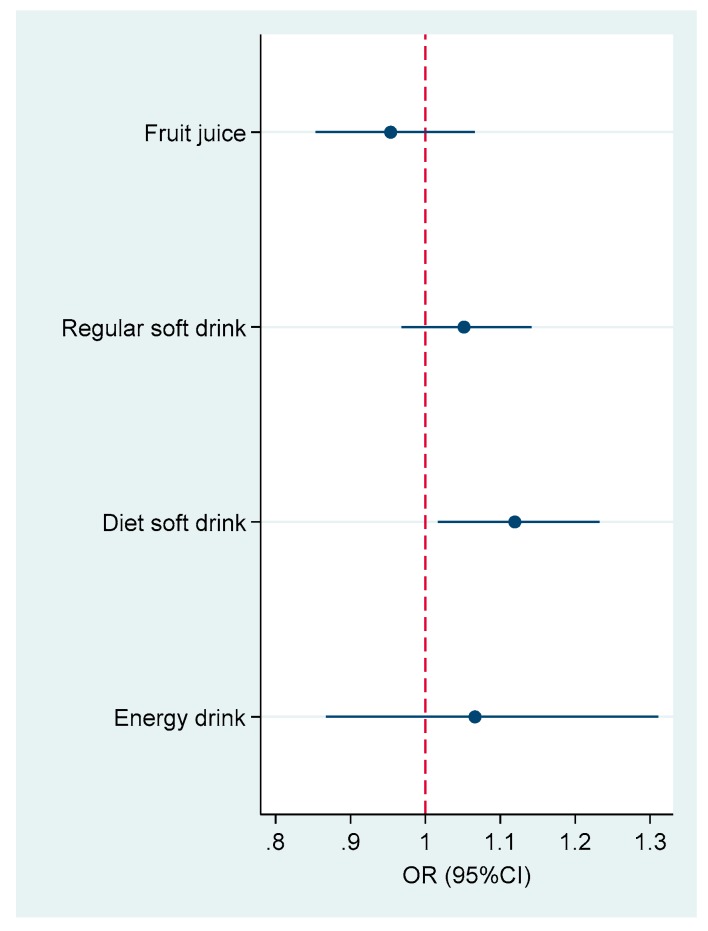
Association between different types of soft drink consumption and asthma. Model adjusted for age, gender, smoking, education, leisure time physical activity, intake of fruit and vegetable, and BMI (normal, overweight and obese). All the soft drinks were mutually adjusted. Soft drinks were used as continuous variables (times/week) in the logistic model.

When we used soft drink as a continuous variable in the multiple variable model, each 1 time/week increase of soft drink consumption was associated with 8.3% (95% CI 3.2–13.7) increased likelihood of having asthma. When all the types of soft drink were mutually adjusted, only diet soft drink (OR 1.12 (95% CI 1.02–1.23)) was statistically association with asthma (Figure 1). Fruit juice consumption had an OR of 0.95 (95% CI 0.85–1.07) for asthma.

### 3.3. Association with FVC and FEV1 

No association between soft drink consumption and FVC was found (Table 3). Consumption of soft drink ≥7 times/week was inversely associated with FEV1. After adjusting for age and gender, compared with non-consumers, FEV1 decreased by 100.7 mL (*p* = 0.057) for those who consumed soft drinks ≥7 times/week. After further adjusting for smoking, education, leisure time physical activity, intake of fruits and vegetables, CRP and BMI, the association was attenuated and became statistically not significant. No dose response relationship was found between soft drink consumption and FEV1. 

**Figure 2:**
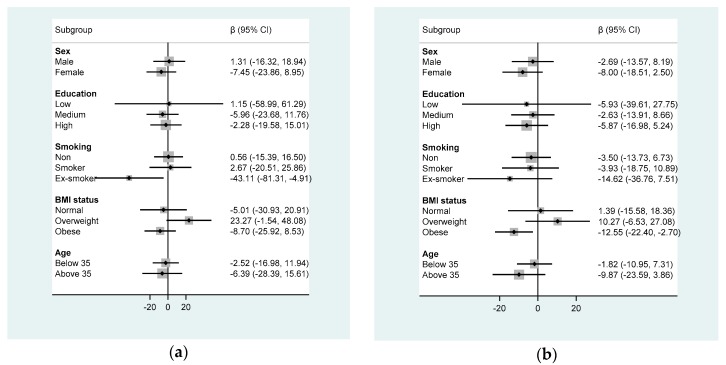
Subgroup analyses of soft drink and lung function. (**a**) FVC (mL); (**b**) FEV1 (mL). Model adjusted for covariates in model 2 of Table 2. Soft drink consumption was used as a continuous variable in the analysis.

In subgroup analyses, soft drink consumption was inversely associated with FVC among ex-smokers (Figure 2). Each 1 time/week increase of soft drink consumption was associated with 43 mL decrease of FVC among ex-smokers. Soft drink was inversely associated with FEV1 among obese participants. The regression coefficient for FEV1 was −12.6 (95% CI −22.4, −2.7) as per 1 time/week increase of soft drink consumption among obese participants.

In sensitivity analysis, we also found that regular soft drink consumption was inversely associated with FEV1 but not with FVC (Figure S2).

## 4. Discussion

In this cross-sectional study, soft drink consumption was positively associated with asthma among Qatari adults. There was a significant association between diet soft drink consumption and asthma. Regular soft drink consumption was associated with decreased FEV1. Fruit juice consumption was positively associated with fruits and vegetables consumption. No association between fruit juice and asthma was found.

Our findings are consistent with previous studies on the association between soft drink consumption and asthma in Australia [16], USA [15,18,20,31], Denmark [32], and the Netherlands [33]. In Australia, using risk behavior surveillance data of 16,907 participants aged 16 years and above, it has been shown that those who consumed more than half a liter of soft drink per day were associated with 26% increased likelihood of having asthma than non-consumers of soft drink [16]. An association between soft drink consumption and asthma has been found among children, adolescents and adults in USA. Using data from the Behavioral Risk Factor Surveillance System in USA, Pak et al. analyzed the association between soft drink consumption and current asthma among high school students [20] and adults. Among 15,960 students (grades 9–12) attending 2009 Youth Risk Behavior Survey, regular soda consumption was positively associated with current asthma [20]. Compared with those who did not drink regular soda, students who drank regular soda two times per day had an OR of 1.28 (95% CI 1.02–1.61) for asthma. Findings from 2013 USA Behavioral Risk Factor Surveillance System suggested that high SSBs consumption was associated with asthma among non-obese participants but not obese participants. Among the non-obese, the OR for having current asthma was 1.66 (95% CI 1.39–1.99) among those who consumed SSBs ≥2 times/day as compared with non-SSBs consumers [15]. In the National Health and Nutrition Examination Survey 2003–2006, excess free fructose beverage consumption was associated with asthma among children aged 2–9 years [18] and chronic bronchitis among adults aged 20–55 years [31]. 

### 4.1. Fruit Juice and Asthma

It has been suggested that intake of high-fructose corn syrup (HFCS) sweetened soft drink was responsible for the increased risk of asthma in USA [34]. In the Framingham Offspring Cohort, those who consumed fruit drink 2–4 times/week were 58% more likely to develop asthma as compared with never/seldom consumers [17]. However, high fruit drink consumption (≥5 times/week) was not associated with the asthma risk [17]. Similarly, fruit juice was positively associated with asthma among children in the Netherland [33]. The “fructositis” hypothesis has been proposed as one of the main mechanisms linking SSB consumption and asthma [34]. However, in the current study, fruit juice was not associated with asthma. The intake of fruit juice was much higher than other types of soft drinks. Interestingly, fruit juice consumption was positively associated with fruit and vegetable consumption as well as leisure time physical activity. In Qatar, fruit juice is often made directly from fresh fruits in the supermarkets. Thus, fruit drink consumption may represent an overall healthy lifestyle and health consciousness. It may partly explain the null association between fruit drink and asthma. 

### 4.2. Diet Soft Drink and Asthma

The association between diet soft drink and asthma is inconclusive [34]. In our study, diet soft drink was positively associated with asthma. Although diet soft drink was positively associated with fruit consumption, it was not related to vegetable consumption (Figure S1) and leisure time physical activity (data not shown). Thus, diet soft drink consumption does not reflect a healthy lifestyle. 

### 4.3. Soft Drink and Lung Function

The association between soft drink consumption and the objective measure of lung function has not been studied extensively. In our study, although there was no clear association between total soft drink consumption and FVC, soft drink consumption was marginally associated with a lower FEV1. Subgroup analyses suggest that soft drink was inversely associated with FVC among ex-smokers. In obese participants, soft drink consumption was associated with a lower FEV1. Among different types of soft drinks, regular soft drink was associated with a lower FEV1. Despite a positive association between diet soft drink and asthma, no association between diet soft drink and FVC and FEV1 was found. Further research is needed to elucidate the inconsistency. 

### 4.4. Potential Mechanisms

In addition to the “fructositis” hypothesis, several other mechanisms may also explain the association between soft drink consumption and asthma including preservatives contained in the soft drink [20,35,36,37], food packaging [38], and inflammation caused by soft drink. Findings from a randomized control trial in Switzerland suggest that consumption of sugar-sweetened beverages increase blood levels of CRP in healthy young men [23]. Soft drink consumption also increases the risk of obesity, which is associated with both inflammation and asthma [39]. In the current study, adjusting for BMI and CRP reduced the OR for asthma associated with high soft drink consumption by 10% (from 2.60–2.33), suggesting that the association between soft drink and asthma was partly mediated by inflammation and obesity. An increasing number of studies suggest that phthalates (a chemical used in soft poly vinyl chloride (PVC) material) are associated with asthma [40]. Soft drinks are often consumed from containers either made of, or lined with, a material that can leach phthalates into the fluid. Human exposure to phthalates is commonly due to ingestion of contaminated food as a result of consuming packaged foods or beverages lined with phthalates [40]. Soft drink consumption was positively associated with urine phthalates levels among Australian men [41]. 

Our study has limitations. Firstly, the food frequency questionnaire (FFQ) only recorded frequency of food intake but not the portion size. Thus, we were unable to estimate the absolute intake of soft drinks and nutrients. We were also not able to separate freshly made fruit juice without food preservatives from those produced in factories with food preservatives. Furthermore, the FFQ was not validated in the study population. Secondly, other environmental variables such as air pollution and incense burning were not considered in this study. Thirdly, asthma was self-reported and the prevalence of asthma was lower in our study as compared with other population based studies in Qatar. The potential selection bias may under or over-estimate the association between soft drink consumption and asthma. Although multivariable models were used, residual confounding cannot be excluded. The sample size and cross-sectional study design limit the generalization and causation. We cannot exclude the possibility of a reverse causation. Those with asthma may have increased their soft drink consumption in order to manage asthma or to take asthma medication. One of the main strengths in our study is the measurement of the lung function, CRP as well as BMI. We were able to adjust for various confounding factors.

## 5. Conclusions

In conclusion, soft drink consumption is adversely associated with lung function and asthma. Promoting healthy fluid drinking behavior should be considered in the prevention and management of asthma. Longitudinal studies on the effects of soft drink consumption and lung function are warranted. 

## Figures and Tables

**Table 1:** Sample characteristics by soft drink consumption among participants attending Qatar Biobank study (*n* = 986).

	None	~1 Time/Week	1–6 Times/Week	≥7 Times/Week	*p* Value
	*n* = 356	*n* = 231	*n* = 269	*n* = 130	
Age	44.6 (12.1)	39.3 (11.1)	36.1 (10.6)	33.2 (8.5)	<0.001
Age groups (years)					<0.001
<40	127 (35.7%)	127 (55.0%)	171 (63.6%)	108 (83.1%)	
40–60	189 (53.1%)	95 (41.1%)	93 (34.6%)	21 (16.2%)	
>60	40 (11.2%)	9 (3.9%)	5 (1.9%)	1 (0.8%)	
Sex					<0.001
Male	142 (39.9%)	125 (54.1%)	150 (55.8%)	81 (62.3%)	
Female	214 (60.1%)	106 (45.9%)	119 (44.2%)	49 (37.7%)	
Education					<0.001
Low	62 (17.4%)	12 (5.2%)	20 (7.5%)	9 (6.9%)	
Medium	86 (24.2%)	55 (23.8%)	79 (29.5%)	53 (40.8%)	
High	208 (58.4%)	164 (71.0%)	169 (63.1%)	68 (52.3%)	
Smoking					<0.001
Non	281 (78.9%)	146 (63.2%)	175 (65.1%)	68 (52.3%)	
Smoker	32 (9.0%)	48 (20.8%)	53 (19.7%)	47 (36.2%)	
Ex-smoker	43 (12.1%)	37 (16.0%)	41 (15.2%)	15 (11.5%)	
BMI (kg/m^2^)	29.5 (5.2)	28.2 (5.2)	28.5 (6.1)	29.2 (6.2)	0.035
BMI status					0.024
Normal	64 (18.0%)	57 (24.7%)	65 (24.2%)	30 (23.1%)	
Overweight	130 (36.5%)	91 (39.4%)	97 (36.1%)	37 (28.5%)	
Obese	147 (41.3%)	71 (30.7%)	78 (29.0%)	51 (39.2%)	
Missing	15 (4.2%)	12 (5.2%)	29 (10.8%)	12 (9.2%)	
CRP levels (%)					0.39
<6 mg/L	245 (68.8%)	149 (64.5%)	178 (66.2%)	82 (63.1%)	
≥6 mg/L	68 (19.1%)	47 (20.3%)	49 (18.2%)	34 (26.2%)	
Missing	43 (12.1%)	35 (15.2%)	42 (15.6%)	14 (10.8%)	
Leisure time physical activity (MET hours/week)	20.8 (38.4)	18.1 (42.7)	17.9 (40.8)	13.4 (22.2)	0.31
FEV1 (mL)	2745.8 (795.5)	2977.7 (751.8)	3156.4 (787.7)	3191.4 (768.2)	<0.001
FVC (mL)	2946.4 (1051.4)	3189.3 (983.5)	3322.0 (1028.8)	3506.8 (1032.4)	<0.001
Asthma	21 (5.9%)	15 (6.5%)	14 (5.2%)	15 (11.5%)	0.098

Data are presented as mean (SD) for continuous measures, and *n* (%) for categorical measures. BMI, body mass index; CRP, C reactive protein; MET, metabolic equivalent; FEV1, forced expiratory volume in one second; FVC, forced vital capacity.

**Table 2:** Odds ratio (95% CI) for asthma by soft drink consumption levels ^1^.

	None	~1 Time/Week	1–6 Times/Week	≥7 Times/Week	*p* Value
Model 1	1.00	1.23 (0.61–2.47)	1.00 (0.48–2.08)	2.51 (1.17–5.36)	0.002
Model 2	1.00	1.23 (0.61–2.51)	1.00 (0.48–2.08)	2.60 (1.20–5.63)	0.001
Model 3	1.00	1.19 (0.58–2.46)	0.94 (0.44–1.98)	2.33 (1.06–5.15)	0.005
Model 4	1.00	1.21 (0.53–2.75)	1.29 (0.59–2.84)	2.64 (1.10–6.36)	0.010

^1^ Model 1 adjusted for age and gender; Model 2 further adjusted for smoking, education, leisure time physical activity, intake of fruit and vegetable; Model 3 further adjusted for BMI (normal, overweight, obese, missing) and CRP (<6, ≥6 mg/L, missing); Model 4 further excluded those first diagnosed asthma below five years of age.

**Table 3:** Regression coefficients (95% CI) for FVC and FEV1 by soft drink consumption levels ^1^.

	None	~1 Time/Week	1–6 Times/Week	≥7 Times/Week	*p* Value
FVC					
Model 1	0.00	−87.1 (−219.7–45.5)	−46.9 (−177.4–83.7)	−12.6 (−178.1–153.0)	0.811
Model 2	0.00	−117.3 (−250.5–15.9)	−54.8 (−185.1–75.6)	−29.1 (−195.5–137.3)	0.813
Model 3	0.00	−116.3 (−249.5–16.8)	−53.1 (−183.7–77.5)	−6.7(−174.0–160.6)	0.889
Model 4	0.00	−126.5 (−261.4–8.5)	−60.0 (−191.6–71.6)	−3.0 (−173.9–167.9)	0.824
FEV1					
Model 1	0.00	−79.0 (−161.9–3.8)	6.4 (−75.2–88.0)	−100.7 (−204.1–2.8)	0.203
Model 2	0.00	−88.3 (−171.9−4.7)	7.8 (−74.0–89.6)	−97.6 (−202.0–6.9)	0.285
Model 3	0.00	−81.7 (−164.9–1.6)	9.5 (−72.2–91.1)	−90.1 (−194.6–14.5)	0.403
Model 4	0.00	−94.0 (−177.7–10.3)	1.4 (−80.2–83.1)	−96.4 (−202.4–9.6)	0.485

^1^ Model 1 adjusted for age and gender; Model 2 further adjusted for smoking, education, leisure time physical activity, intake of fruit and vegetable; Model 3 further adjusted for BMI (normal, overweight, obese, missing) and CRP (<6, ≥6 mg/L, missing); Model 4 further excluded those first diagnosed asthma below five years of age. FVC, forced vital capacity; FEV1, forced expiratory volume in one second.

## Supplementary Materials

The following are available online at https://www.mdpi.com/2072-6643/11/3/606/s1, **Figure S1.** Association between different types of soft drink consumption and fruit and vegetable consumption. **Figure S2.** Association between different types of soft drink consumption and lung function.
