# Supplementary material for: Association between Soft Drink Consumption and Asthma among Qatari Adults
Source: Nutrients. 2019 Mar 13;11(3):606. doi: 10.3390/nu11030606 (PMC6471171; doi:10.3390/nu11030606)
Supplement: Supplementary file 1 [file nutrients-11-00606-s001.pdf]

Figure S1. Association between different types of soft drink consumption and fruit and vegetable consumption.

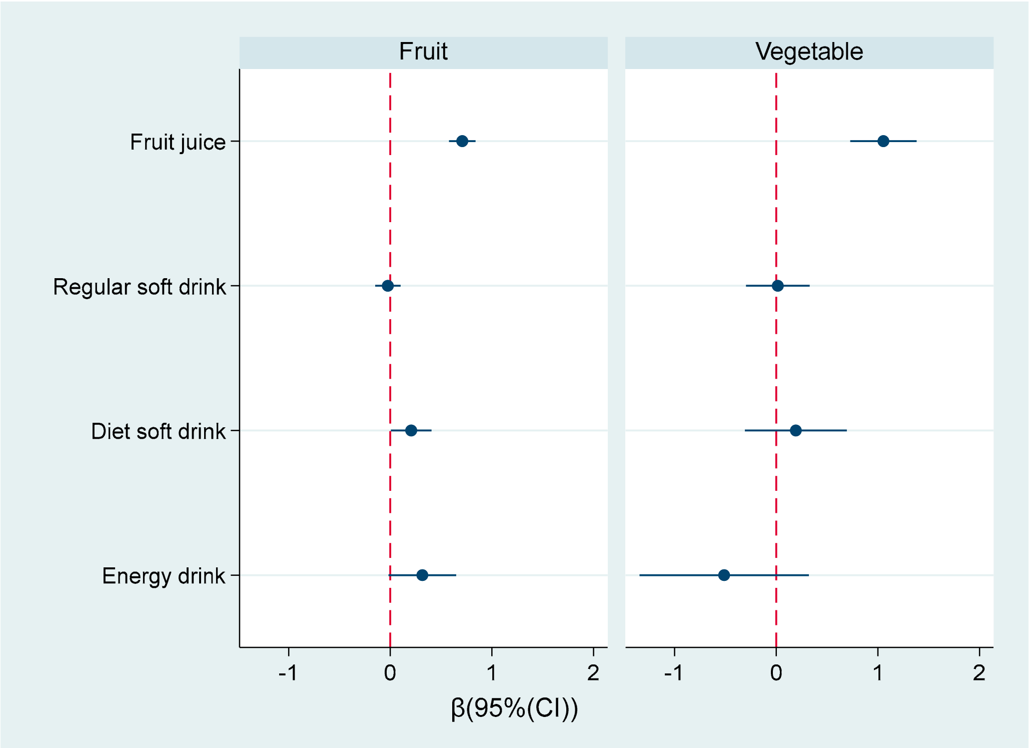

Values are adjusted for age and gender. All the different types of soft drink were mutually adjusted.

Figure S2. Association between different types of soft drink consumption and lung function.

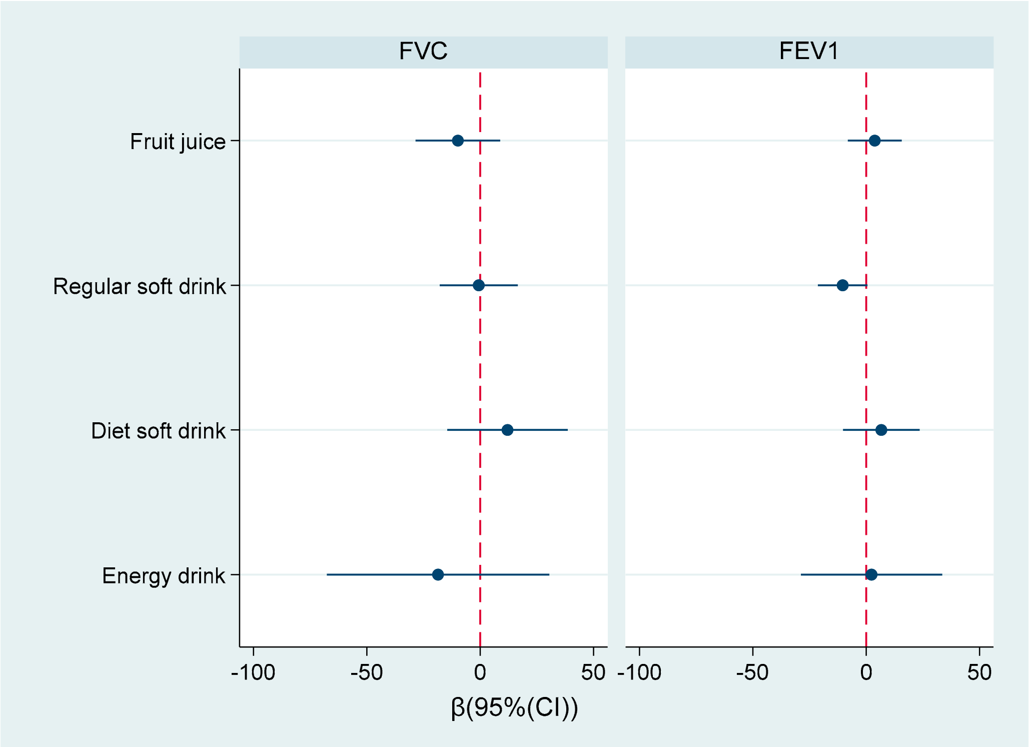

Values adjusted for age, gender, education, physical activity, smoking, BMI, and intake of fruit and vegetables. Different types of soft drink were mutually adjusted as times/week. FVC and FEV1 were presented as mL.
